# Implicit Attitudes towards People with Intellectual Disabilities: Their Relationship with Explicit Attitudes, Social Distance, Emotions and Contact

**DOI:** 10.1371/journal.pone.0137902

**Published:** 2015-09-14

**Authors:** Michelle Clare Wilson, Katrina Scior

**Affiliations:** 1 Department of Plastic and Reconstructive Surgery, Royal Free Hospital, London, United Kingdom; 2 Research Department of Clinical Educational & Health Psychology, University College London, London, United Kingdom; University of Perugia, ITALY

## Abstract

Implicit attitude research has expanded rapidly over the last decade and is seen as very promising as it counters biases present in much attitude research such as social desirability. However, most research in the area of intellectual disabilities has focused on explicit attitudes alone. This study examined implicit attitudes to this population and also examined their association with emotional reactions and contact, which have previously been found to have a significant influence on attitudes and stigma. A web based survey consisting of a single target Implicit Association Test, measures of explicit attitudes, social distance, and emotional reactions towards and contact with individuals with intellectual disabilities was completed by 326 adult UK residents. Implicit attitudes were not significantly associated with explicit attitudes, social distance or emotional reactions. Instead there were small to moderate associations between emotional reactions and explicit attitudes and social distance. Implicit attitudes did not vary according to participants’ level of contact with individuals with intellectual disabilities, type of the contact relationship (voluntary versus involuntary), gender or educational attainment. In contrast, these participant characteristics did affect explicit attitudes and social distance. Implicit attitudes towards individuals with intellectual disabilities were somewhat negative and, unlike explicit attitudes and stigma, did not vary according to participant demographics or contact. As they may have a negative impact on the lives of people with intellectual disabilities, implicit attitudes merit increased attention in research and interventions in the intellectual disabilities field.

## Introduction

The fight for equal rights and inclusion of people with intellectual disabilities has led to considerable changes to policy and service provision over the last 30 years. The most notable changes consist of the closure of long stay institutions in favour of community based care and the move away from segregated learning environments to inclusive education in many countries globally. Notwithstanding these changes, this population remains one of the most socially excluded and vulnerable [1–2]. Despite increased physical integration, lack of genuine social inclusion has been identified as a prime concern [3–5]. Such reports point to a need to increase our understanding of the societal context, and to identify methods to measure community responses to this population in a reliable and valid fashion.

### Attitudes and attitude formation

Attitudes refer to the extent of an individual’s favour or disfavour towards a particular attitude object [6]. Two distinct types of attitude (explicit and implicit) have been identified. Explicit attitudes are evaluations which are consciously accessible and controllable, whereas implicit attitudes are evaluations which are automatically activated and occur without effort or intention [7]. Theorists have suggested various dual-processing models to try and explain how these different types of attitudes influence individuals in different circumstances (e.g. [8–10]). For the most part, the theories appear to share a common principle: information processing in relation to an attitude object occurs in two different ways depending on whether an individual has the cognitive capacity, motivation and time available to consciously consider the attitude object or not. A dual-process type model for understanding the influence of attitudes on behaviour was proposed by Strack and Deutsch [11]. It suggests there are two information processing systems: a reflective processing system (which allows for conscious consideration of relevant information) and an impulsive processing system (which is always activated and does not require very much cognitive capacity). It is suggested that these processes are not independent of each other but operate in parallel, with factors such as cognitive demand at the time, determining which process ultimately influences behaviour [11]. They note that the reflective system is likely to influence behaviour when an individual has time to consider the value and consequences of their behaviours and the motivation to do so [11]. This is similar to what other researchers have referred to as deliberate behaviours influenced by explicit attitudes [12]. Furthermore, it is suggested that the impulsive system influences behaviours when resources are not available (e.g. time to consider the consequences of one’s behaviour) and/or motivation to do so is low [11] and links closely to the idea of spontaneous behaviours influenced by implicit attitudes [12].

There have been many studies which have explored implicit and explicit attitudes over the last few decades with a variety of measures now available to do so e.g. self-report questionnaires to explore explicit attitudes and response speed tasks to explore implicit attitudes [13]. It is suggested that self-report type measures that explore explicit attitudes tap into conscious evaluations that the individual believes to be true, whereas implicit attitude measures tap into more automatic associations that are not consciously accessible [13]. The relationship between measures of implicit and explicit attitudes is complex, with some researchers suggesting that factors such as social desirability, availability of cognitive resources and importance of the attitude held influence the degree of association between them [14]. It is also suggested that the extent to which an individual elaborates on their explicit attitude will also effect the association these have with implicit attitudes—more elaboration leading to less association between implicit and explicit attitudes [13]. Overall, given the complexity of these processes and constructs, it seems reasonable that to ensure a comprehensive exploration of attitudes held, both implicit and explicit attitudes should be explored.

Furthermore, different processes are thought to be involved in the formation of these two types of attitude. According to the Value-Account model [15], implicit attitudes are formed by unintentional or automatically activated processes which require limited cognitive resources; whereas explicit attitudes are formed by processes controlled by the individual and consume cognitive resources. This model further posits that explicit and implicit attitudes are represented in memory in different ways. Implicit attitudes consist of value accounts, i.e. accumulations of implicit value charged information of an attitude object. As such they are formed using a weighted summation rule, that is, they are based on the aggregation of all the information pertaining to a particular attitude object that is capable of eliciting an affective reaction (positive or negative). As such, each time value-charged information relating to a particular attitude object is encountered, the new information increments or decrements the already established value-account pertaining to the attitude object [15]. This process is not consciously controlled by the individual and happens automatically upon encountering the value-charged information. The intensity of these value accounts is expressed through the affective system and is accessible immediately when the attitude object is activated in memory [15].

Explicit attitudes are represented as summary evaluations which are based on information pertaining to particular value charged episodes or events relating to an attitude object. This information is expressed as a global representation of the attitude object in the form of a summary evaluation. As such explicit attitudes are formed using a weighted averaging rule, that is, they are produced by taking an average of the weighted sample of evaluations of the attributes of a particular attitude object [7]. That is, information stored in memory relating to a particular attitude object is consciously accessed and considered alongside new information (if available), to produce an average evaluation / judgement of the attitude object [15]. Explicit attitudes are not directly influenced by affective reactions as summary evaluations are subject to controlled processes. Therefore only if the affective reaction is actively appraised can it have an influence on explicit attitude formation [15]. This model was supported by Prestwich et al. [7] who found that implicit attitudes were associated with quantity of contact with an out-group (in line with the summation rule) and that quality of contact was associated with explicit attitudes (in line with the averaging rule).

### Social distance

Social distance refers to the willingness an individual has to engage with a member of another group in situations of varying degrees of intimacy [16]. Researchers have used social distance as a measure of external stigma towards various stigmatised populations, including individuals with mental health problems (see [17] for a review), and people with intellectual disabilities [18–19].

### Association between contact, attitudes and social distance

The contact hypothesis [20] suggests that contact between members of different social groups can help reduce prejudice. Support for the association between contact and attitudes has been reported in numerous studies, with researchers finding that contact in various forms (e.g. voluntary, intimate, direct, and indirect) can help to improve prejudiced attitudes [21–23]. This extends to individuals with intellectual disabilities, with research suggesting that those who have more contact with individuals with intellectual disabilities hold more positive explicit attitudes towards them than those with less frequent contact [24–27]. Similarly, it has been reported that contact can reduce the desire for social distance from members of out-groups [17–18].

### The role of emotions in the contact-attitude relationship

Emotions are thought of as one of the three key components of attitudes [28]. Numerous studies have identified the important influence of emotions on attitudes towards a variety of social groups and situations, including interracial interactions [7], inter-group friendships [29–30] and interactions between individuals with and without disabilities within a school context [31]. Emotional reactions play an important role in the contact-attitude relationship [32–33], as well as desire for social distance from an out-group [34]. Increased contact is associated with lower levels of inter-group anxiety and more positive attitudes towards the out-group [32–33] and increased familiarity with an out-group being associated with less negative emotional reactions towards an out-group and a lower desire for social distance from them [34].

Studies investigating emotional reactions towards individuals with intellectual disabilities have mostly focused on emotional reactions of care staff to challenging behaviours [35–37]. Research that either investigates emotional reactions not specifically related to episodes of challenging behaviour or that explores the emotional reactions of members of the general public towards this population is lacking.

### The present study

The reliance of most attitudinal research in the intellectual disability field on self-reported explicit attitudes poses significant risks to validity given that factors such as respondent reactivity may influence reported attitudes [38]. Explicit attitudes have also been shown to vary according to participant demographics e.g. individuals with higher educational attainments tend to express more positive explicit attitudes towards individuals with disabilities than those with lower educational attainments (e.g. [2, 27]) and women often report more positive explicit attitudes towards individuals with disabilities than men (e.g. [2, 39–40]). Some studies have also suggested that gender and educational attainment can have an effect on social distance (e.g. [18, 41]). On the other hand, implicit attitude research has suggested that these demographic variables often do not influence implicit attitudes, e.g. several studies found there were no differences between men’s and women’s implicit attitudes towards individuals with disabilities [42–44]. Measurement of less consciously controllable implicit attitudes may provide a more accurate reflection of attitudes and may elucidate if factors such as participant demographics influence implicit attitudes towards individuals with intellectual disabilities. Implicit attitude research often involves the use of the Implicit Association Test (IAT) [45]. While in the original IAT implicit attitudes towards one attitude object are compared to another, the Single Target IAT (ST-IAT) [46–48], used in this study, allows measurement of attitudes towards one attitude object alone, and makes interpretation of scores less ambiguous.

Exploration of explicit attitudes and social distance in parallel was conducted to ascertain to what extent these diverge from implicit attitudes. While the relationship between contact and explicit attitudes has been explored fairly extensively [24–27, 49], the relationship between implicit attitudes and contact with individuals with intellectual disabilities to date has not been explored. Lastly, previous studies have suggested that emotional reactions mediate the relationship between contact and attitudes / social distance, yet there is a distinct lack of studies which examine emotional reactions towards individuals with intellectual disabilities. Hence it seemed pertinent to examine their role as part of the present study.

In examining the relationship between the constructs in question, we put forward the following hypotheses: (1) the relationship between implicit and explicit attitudes towards individuals with intellectual disabilities would be non-significant; (2) the relationship between implicit attitudes and social distance would be non-significant; (3) the association between explicit attitudes and social distance would be significant; (4) in support of the Value-Account model [15], there would be a stronger association between implicit attitudes and emotional reactions than between explicit attitudes or social distance and emotional reactions; and implicit attitudes would vary according to amount of contact to a greater extent than explicit attitudes or social distance; finally, (5) explicit attitudes and social distance, but not implicit attitudes, would differ by participant gender, educational attainment, and type of contact (voluntary versus involuntary).

## Method

### Pilot study

A pilot study was undertaken to identify a set of words to be used in the ST-IAT. Forty individuals were presented with a list of 15 terms associated with the category of ‘learning disabilities’, the term most commonly used in the UK to indicate intellectual disability (dependent, mental handicap, impaired, slow learner, child-like, special needs, innocent, vulnerable, mentally retarded, incapable, delayed development, not quite human, incompetent, retarded and deviant). Participants rated how representative these were of individuals with ‘learning disabilities’ on a scale from 0 (completely unrepresentative) to 10 (completely representative). The five terms rated the most representative (dependent, mental handicap, slow learner, impaired, special needs) were used in the ST-IAT.

### Participants

Full data sets were collected from a convenience sample of 326 adult UK nationals / residents. The mean age of the sample was 34.76 years (SD = 12.07). Participant demographic information is presented in Table 1. With regards to inclusion / exclusion criteria, this study was open to anyone aged 18 years or older who had been living in the UK for at least three years.

**Table 1:** Participant demographics.

		*N*	%
Gender	Male	106	32.5
	Female	220	67.5
Ethnicity	White British	275	84.4
	White other	24	7.4
	Asian	17	5.1
	Black African/Caribbean	2	0.6
	Other	8	2.5
Education level	To age 16 (e.g. GCSE)	30	9.2
	To age 18 (e.g. A Levels)	41	12.6
	University degree	105	32.2
	Post-graduate	150	46.0
Know someone with ID	Yes	199	61.0
	No	127	39.0
Nature of relationship^a^	Voluntary	117	36.1
	Involuntary	80	24.7
	Not applicable	127	39.2
Frequency of contact	Not applicable	143^b^	43.9
	Infrequently	106	32.5
	1 or 2 times per month	32	9.8
	1 or 2 times per week	20	6.1
	Daily or almost daily	25	7.7

^a^The total number for this category is 324 as two participants did not state what their relationship with individuals with ID was.

^b^Some participants reported knowing an individual with an ID but were no longer in contact with them; hence the frequency seen was “not applicable”.

### Measures

The measures detailed below were included to assess implicit attitudes, explicit attitudes, social distance, emotional reactions and contact.

#### Single Target Implicit Association Test (ST-IAT)

The IAT [45] measures the relative strength of association between pairs of concepts by presenting four different groups of images/words on the screen [50]. Participants categorise the presented image/word using one of two keyboard keys. Two of these groups are target concepts (e.g. disabled and non-disabled) and the remaining two are attribute concepts (e.g. pleasant and unpleasant).

In the ST-IAT, implicit attitudes to a single target or category are measured, along with the two attribute concepts (e.g. disabled, good and bad) [46–48]. The ST-IAT was deemed more appropriate for this study, as there is no obvious complementary target concept to use alongside that of intellectual disability. It consists of five blocks of trials: in Block 1 (20 trials) participants practiced categorising the two sets of attribute category words (five ‘pleasant’ words: happiness, laughter, joyful, rainbow, sunshine and five ‘unpleasant’ words: sickness, hatred, disease, terrible, poison) using two keyboard keys, e.g. ‘pleasant’ paired to the left key and ‘unpleasant’ to the right key. The words were taken from stimuli lists used in previous studies (http://faculty.washington.edu/agg/bytopic.htm). In Block 2 (20 trials) the five words representing the target category ‘learning disabilities’ was added to one response key and participants practiced categorising all three sets of words. Block 3 was identical except the number of trials was increased to 40. In Block 4 (20 trials) the category ‘learning disabilities’ was swapped to the opposite response key; participants practiced categorising all three sets of words with this new positioning. Block 5 was identical to block four except an increase in the number of trials to 40. Blocks 3 and 5 were used as the actual trial blocks for the purposes of scoring.

It is the difference in response time to the different pairings that gives an indication of participants’ implicit attitudes. If participants are quicker categorising words when ‘learning disabilities’ and ‘pleasant’ are paired, this indicates positive implicit attitudes. Conversely, if they are quicker categorising words when ‘learning disabilities’ and ‘unpleasant’ are paired, this indicates negative implicit attitudes.

Congruent and incongruent blocks, that is whether the categories ‘learning disability’ and ‘unpleasant’ or ‘pleasant’ were paired to the same key, were counterbalanced between participants and the improved scoring algorithm [51] was used to obtain a single implicit attitude score for each participant. Accordingly, for the trial blocks 3 and 5, the average response time for the congruent block was subtracted from the average response time of the incongruent block, before dividing this value by the standard deviation of all correct response times. The final resulting value indicates the participant’s implicit attitude. Values below zero are interpreted as indicating a negative implicit attitude, values above zero a positive implicit attitude; the greater the value, the stronger the implicit attitude. Scores range from 2 to -2 and in line with previous research using the Implicit Association Test (see [52]), the strength and direction of the implicit attitude measured by the ST-IAT was assessed using the scores detailed in Table 2.

**Table 2:** ST-IAT score descriptions.

Score	Description	
-2 to -0.65	Strong negative	
-0.65 to -0.36	Moderate negative	
-0.36 to -0.15	Slight negative	
-0.15 to 0.15	No preference / neutral	
0.15 to 0.36	Slight positive	
0.36 to 0.65	Moderate positive	
0.65 to 2	Strong positive	
**Score**		**Description**
-2 to -0.65		Strong negative
-0.65 to -0.36		Moderate negative
-0.36 to -0.15		Slight negative
-0.15 to 0.15		No preference / neutral
0.15 to 0.36		Slight positive
0.36 to 0.65		Moderate positive
0.65 to 2		Strong positive

Although these criteria were used to assess the implicit attitudes measured in previous studies using the IAT, it must be noted that there has been critique of this procedure as these score ranges are not based on specific behavioural observations that quantify them more accurately [53]. However given that these are the available criteria to assess the scores produced by the ST-IAT, they were used in the study. Internal consistency was assessed by calculating Cronbach α of the difference scores for the 40 test trials from blocks 3 and 5, the score being .70. This suggests good internal consistency of the ST-IAT.

#### Community Living Attitudes Scale–ID Version (CLAS-ID)

The CLAS-ID [54] assesses explicit attitudes towards individuals with intellectual disabilities on four subscales (Empowerment, Exclusion, Sheltering and Similarity). For this study the 17 item short version, developed by the original authors in parallel with the full 40 item version [54], was used. Participants rated their agreement with each item from 1 (disagree strongly) to 6 (agree strongly). Higher scores on the Empowerment and Similarity subscales suggest more inclusion friendly attitudes, whereas higher scores on the Exclusion and Sheltering subscales suggest less inclusion friendly attitudes. Internal consistency of the subscales was moderate to good (Empowerment: Cronbach α = .75; Sheltering: Cronbach α = .64; Exclusion: Cronbach α = .78; Similarities: Cronbach α = .79).

#### Social Distance

Two measures of social distance were included: the Social Distance subscale of the Intellectual Disability Literacy Scale (IDLS) [55] and the social distance subscale of the Multidimensional Attitude Scale on Mental Retardation (MASMR) [56]. Both assess a respondent’s willingness to engage in contact with individuals with ID in situations of varying degrees of intimacy. The MASMR social distance subscale has been used frequently over the years including recent large scale population studies [18]. The IDLS social distance subscale is derived from research on mental health stigma, is briefer than its MASMR parallel, and has good psychometric properties across a wide range of cultural groups [55]. By including both measures, the current findings can be evaluated in light of previous studies which have used these measures.

#### Social distance subscale of the IDLS

The social distance subscale of the IDLS [55] consists of five items rated on a 7 point Likert scale (1 = disagree strongly to 7 = strongly agree). Participants completed these items in relation to a young man with moderate to severe intellectual disabilities presented in a vignette (see S1 Appendix), that had been evaluated by trained clinicians for its accuracy of depiction prior to its use in this study.

#### Social distance subscale of the MASMR

The social distance subscale of the MASMR [56] contains eight statements rated on a 4 point Likert scale (1 = strongly disagree to 4 = strongly agree).

For both scales scores are reversed and a mean score is calculated, with higher scores indicating a greater desire for social distance. Internal consistency of both scales was very good (Cronbach α = .87 for the IDLS, and .85 for the MASMR subscales).

#### Emotional Reactions to Mental Illness Scale (ERMIS)

The ERMIS [57] consists of 9 items designed to measure emotional reactions on three dimensions (fear, compassion and anger), using a 7 point Likert scale (1 = disagree strongly to 7 = agree strongly). This scale was originally developed to assess emotional reactions towards individuals with mental illness, but has recently been used to assess affective responses to individuals with intellectual disabilities [58]. For the purposes of this study, participants completed these items in response to aforementioned vignette (S1 Appendix).

A mean score for each subscale is calculated; higher scores indicate greater endorsement of the respective emotion. Internal consistencies of the subscales were calculated, with the Fear (Cronbach α = .76) and Compassion (Cronbach α = .76) subscales showing good internal consistency. For the Anger subscale, inclusion of all three items yielded a Cronbach α of .56. Exclusion of item 3 (*I feel angry towards him*), resulted in an improved α value of .76; hence all subsequent analyses were carried out using only the two remaining Anger items.

#### Contact

Participants indicated whether they had prior contact with someone with an intellectual disability, the type of contact relationship, frequency of contact, and its closeness (rated on a 9-point Likert scale from 1 = not at all close to 9 = extremely close). In case of several prior contact relationships, they were asked to rate the closest one.

### Procedure

Ethical approval was granted by the second authors’ institutional ethics committee (University College London Research Ethics Committee). In this web based study, an invitation to participate in the study was distributed by email to social and work contacts of the authors, through postings on Facebook, and advertisements on web discussion forums (not specific to disability). Potential participants were presented with information about the study, including a definition of ‘learning disability’ to counter misconceptions about the meaning of the term, before proceeding to the survey’s introduction page. The introduction page clearly stated that participation was voluntary, that participants had the right to withdraw from the study at any time and that by clicking on the link to continue with the study, participants were providing consent for their data to be used for the purposes of the study. This procedure was approved by the second author’s institutional ethics committee (University College London Research Ethics Committee). An incentive in the form of a prize draw for a cash prize was offered. The presentation order of the ST-IAT and self-report measures was counterbalanced between participants.

### Data analysis

The data was analysed using SPSS for Windows version 21. Two participants were excluded as they had more than 20% errors on the ST-IAT. Participants’ relationships with individuals with intellectual disabilities were coded as either voluntary (friend, spouse, work with individuals with intellectual disabilities e.g. support worker), involuntary (family member, neighbour, colleague) or ‘not applicable’ in case of no reported contact. As not all participants provided full data sets, sample sizes vary for the different analyses.

## Results

Examination of ST-IAT scores (using the criteria stated in the method section for interpreting these scores) suggested on average participants showed a slight negative bias towards individuals with intellectual disabilities (*M* = -0.18, SD = 0.34); *t* (328) = -9.81, *p*< 0.001, with a 95% confidence interval ranging from -0.22 to -0.15 (see Table 3).

**Table 3:** Means and standard deviations for implicit attitudes, explicit attitudes, social distance and emotional reaction scores.

Measure	Subscale	*N*	*M*	SD
ST-IAT	d-score^a^	329	-0.18	0.34
IDLS	Social Distance^b^	338	2.52	1.17
MASMR	Social Distance^c^	338	1.42	0.43
CLAS-ID^d^	Empowerment	338	4.64	0.83
	Exclusion	338	1.51	0.71
	Sheltering	338	3.30	0.82
	Similarities	338	5.51	0.64
ERMIS^b^	Compassion	338	4.65	1.43
	Anger	338	1.40	0.77
	Fear	338	1.82	1.05

^a^Scores range from -2 to 2.

^b^Scores range from 1 to 7.

^c^Scores range from 1 to 4.

^d^Scores range from 1 to 6.

The distribution of participants’ ST-IAT scores is detailed in Table 4.

**Table 4:** Distribution of participants across ST-IAT score ranges.

	*N*	%
Strong negative (-2 to -0.65)	25	7.60
Moderate negative (-0.65 to -0.36)	89	27.05
Slight negative (-0.36 to -0.15)	70	21.28
No preference / neutral (-0.15 to 0.15)	85	25.84
Slight positive (0.15 to 0.36)	44	13.37
Moderate positive (0.36 to 0.65)	12	3.65
Strong positive (0.65 to 2)	4	1.21

In contrast, scores on the Empowerment, Exclusion and Similarities subscales of the CLAS-ID were suggestive of highly positive explicit attitudes (Table 3). To a somewhat lesser extent, scores on the Sheltering subscale were also suggestive of positive explicit attitudes. Participants reported a low desire for social distance from individuals with intellectual disabilities on both social distance measures used. They tended to respond with low levels of anger and fear but high levels of compassion towards the individual depicted in the vignette (Table 3).Therefore on the whole, participants showed a slight negative implicit bias yet positive explicit attitudes towards individuals with intellectual disabilities.

### Relationships between attitudes, social distance and emotional reactions

Each hypothesis was examined in turn. For hypotheses one to four the significance level was Bonferroni adjusted to account for multiple correlations (*p*-value = .001, i.e.: .05 / 45). Kendall’s Tau-b correlation coefficients for the associations between implicit attitudes, explicit attitudes, social distance and emotional reactions are presented in Table 5.

**Table 5:** Kendall’s Tau-b correlation coefficients for implicit attitudes, explicit attitudes, social distance and emotional reactions.

Measure	1	2	3	4	5	6	7	8	9
1) ST-IAT	—								
2) ERMIS Fear	-.07	—							
3) ERMIS Compassion	-.04	.19**	—						
4) ERMIS Anger	-.09	.50**	.09	—					
5) CLAS-ID Empowerment	.002	-.33**	-.13*	-.27**	—				
6) CLAS-ID Exclusion	-.07	.34**	.07	.36**	-.38**	—			
7) CLAS-ID Sheltering	.01	.19**	.27**	.15**	-.33**	.16**	—		
8) CLAS-ID Similarities	.01	-.30**	-.15**	-.28**	.45**	-.43**	-.23**	—	
9) IDLS Social Distance	-.12*	.38**	.06	.33**	-.39**	.41**	.22**	-.37**	—
MASMR Social Distance	-.06	.39**	.05	.35**	-.40**	.42**	.23**	-.42**	.57**

*N* = 324.

*p< .005.

**p< .001.

#### Relationship between implicit attitudes, explicit attitudes and social distance

In line with hypotheses one and two, the relationships between implicit attitudes, explicit attitudes and social distance were weak overall. No significant associations were observed between implicit attitudes and explicit attitudes or social distance measured on the MASMR. A small association between IDLS social distance scores and implicit attitudes was observed (Kendall’s Tau b = -.12, *N* = 324, *p* = 0.002), which did not meet the adjusted significance level of 0.001. In line with hypothesis three, significant small to moderate associations were found between all CLAS-ID subscales and both social distance measures. On the whole, these correlations indicate that those who hold more positive explicit attitudes had a lower desire for social distance.

#### Relationship between implicit attitudes, explicit attitudes, social distance and emotional reactions

Against our predictions (hypothesis four), implicit attitudes and emotional reactions were not correlated. All three emotional reactions subscales correlated with CLAS-ID Empowerment, Sheltering and Similarities scores; the Fear and Anger subscales correlated with CLAS-ID Exclusion scores. This suggests that those who held more positive explicit attitudes express less fear and anger, but also less compassion. Lower ratings of fear and anger were in turn associated with lower social distance (Table 5).

### Differences in implicit attitudes, explicit attitudes and social distance scores by frequency of contact

Descriptive data according to frequency of contact are presented in Table 6.

**Table 6:** Means and standard deviations of implicit attitudes, explicit attitudes and social distance for varying levels of contact frequency.

			Measures													
			ST-IAT D-score^a^		IDLS Social Distance^b^		MASMR^c^ Social Distance		CLAS-ID Empowerment^d^		CLAS-ID Exclusion^d^		CLAS-ID Sheltering^d^		CLAS-ID Similarities^d^	
		*N*	*M*	SD	*M*	SD	*M*	SD	*M*	SD	*M*	SD	*M*	SD	*M*	SD
Know person with ID	Yes	197	-.015	0.33	2.30	1.06	1.36	0.41	4.66	0.82	1.43	0,65	3.32	0.82	5.57	0.61
	No	127	-0.22	0.34	2.85	1.24	1.52	0.44	4.57	0.86	1.64	0.78	3.28	0.83	5.41	0.69
Contact Frequency	Not applicable	143	-0.21	0.34	2.79	1.23	1.50	0.43	4.67	0.86	1.60	0.77	3.28	0.81	5.42	0.68
	Infrequent	106	-0.18	0.32	2.50	1.10	1.42	0.46	4.57	0.84	1.50	0.73	3.36	0.84	5.52	0.67
	1 or 2 times per month	32	-0.09	0.31	2.06	0.95	1.32	0.35	4.75	0.70	1.35	0.54	3.22	0.83	5.63	0.55
	1 to 2 times per week	20	-0.03	0.32	2.07	1.00	1.34	0.45	4.80	0.71	1.45	0.67	3.19	0.83	5.55	0.55
	Daily or almost daily	25	-0.18	0.34	1.90	0.93	1.22	0.28	4.96	0.71	1.30	0.48	3.38	0.81	5.74	0.42

Contrary to predictions (hypothesis four), implicit attitudes did not differ between participants who reported prior contact with someone with intellectual disabilities and those who did not, *t*(322) = 1.74, *p* = .82, yet IDLS (*U* = 16 029.5, z = 4.10, *p* < .001) and MASMR Social Distance scores (*U* = 15 656, z = 3.68, *p* < .001) differed between the two groups. Implicit attitudes did also not differ by frequency of contact, *F* (4, 319) = 1.94, *p* = 0.10, yet Kruskall-Wallis tests suggested differences in IDLS (χ^2^ = 24.01, *p*< .001) and MASMR Social Distance scores (χ^2^ = 17.01, *p* = .002) with varying frequency of contact. Of note, mean ST-IAT scores became less negative with increasing contact up to the point of frequent contact (1x or 2x per week), but scores of those reporting daily contact were equivalent to those who only had infrequent contact. IDLS social distance scores differed between those with daily or almost daily contact and those with no contact (χ^2^ = 72.22, *p* = .002), and between those with contact once or twice monthly and those with no contact (χ^2^ = 57.28, *p* = .02). MASMR Social Distance scores differed between individuals with daily or almost daily contact and those with no contact (χ^2^ = 68.04, *p* = .01). For all of these findings, individuals with more frequent contact expressed lower desire for social distance than those with less frequent contact.

In line with predictions (hypothesis four), CLAS-ID Empowerment (*U* = 12 023, z = -0.74, *p* = .46) and Sheltering (*U* = 12 199, z = -0.53, *p* = .60) scores did not differ between participants with or without prior contact. However, Exclusion (*U* = 14 775, z = 2.71, *p* = .01) and Similarities (*U* = 10 457, z = -2.73, *p* = .01) scores differed between these two groups. Kruskal-Wallis analyses showed no differences by frequency of contact in CLAS-ID Empowerment (χ^2^ = 5.85, *p* = .20), Sheltering (χ^2^ = 1.50, *p* = .83), Exclusion (χ^2^ = 5.41, *p* = .23), or Similarities scores (χ^2^ = 9.60, *p* = .05), once the significance level was Bonferroni adjusted.

### Implicit attitudes, explicit attitudes and social distance scores by participants’ gender, education level and contact relationship

Descriptive data according to participant characteristics are presented in Table 7. Due to small group sizes, participants educated to age 16 and 18 were combined for the purposes of analysis.

**Table 7:** Means and standard deviations for implicit attitudes, social distance and explicit attitudes on various participant characteristics.

			Measure													
			ST-IAT D-score^a^		IDLS Social Distance^b^		MASMR^c^ Social Distance		CLAS-ID Empowerment^d^		CLAS-ID Exclusion^d^		CLAS-ID Sheltering^d^		CLAS-ID Similarities^d^	
		*N*	*M*	SD	*M*	SD	*M*	SD	*M*	SD	*M*	SD	*M*	SD	*M*	SD
Gender	Male	106	-0.17	0.35	2.86	1.25	1.53	0.46	4.48	0.83	1.73	0.80	3.33	0.80	5.38	0.68
	Female	220	-0.18	0.33	2.35	1.09	1.37	0.40	4.70	0.83	1.40	0.63	3.29	0.84	5.57	0.62
Education level	Up to age 18 years	71	-0.11	0.30	2.75	1.36	1.59	0.57	4.30	1.03	1.75	0.96	3.68	0.84	5.22	0.88
	Graduate	105	-0.17	0.34	2.48	1.25	1.40	0.40	4.63	0.80	1.52	0.72	3.16	0.84	5.47	0.60
	Postgraduate	150	-0.22	0.35	2.43	0.98	1.36	0.35	4.78	0.71	1.39	0.52	3.23	0.76	5.67	0.48
Nature of relationship	Voluntary	117	-0.15	0.34	2.22	1.02	1.35	0.41	4.72	0.87	1.43	0.65	3.27	0.86	5.61	0.62
	Involuntary	80	-0.15	0.31	2.42	1.13	1.37	0.42	4.57	0.75	1.44	0.67	3.37	0.76	5.50	0.61

In line with hypothesis five, implicit attitudes did not differ between men and women (*t* (322) = -0.34,*p* = .74), between those in voluntary or involuntary contact relationships (*t* (193) = -.011,*p* = .91), nor by educational attainment, *F* (2, 321) = 2.88, *p* = .06. Explicit attitudes, as predicted, were affected by participant characteristics: CLAS-ID Empowerment (U = 9753, z = -2.40, *p* = .02), Exclusion (*U* = 14 970, z = 4.37, *p*< .001) and Similarities (*U* = 9169, z = -3.24, *p* = .001) scores, but not Sheltering scores (*U* = 12 078, z = 0.53, *p* = .60), differed between men and women, with women showing more positive explicit attitudes than men. Kruskall-Wallis analyses showed more positive explicit attitudes for those with higher educational attainments: Empowerment: χ^2^ = 11.97, *p* = .003, Sheltering χ^2^ = 17.30, *p*< .001, Exclusion: χ^2^ = 6.00, *p* = .05 and Similarities: χ^2^ = 19.33, *p*< 0.001. Empowerment and Exclusion scores were more positive for postgraduates than those educated to age 18 (χ^2^ = -46.52, *p* = .002 and χ^2^ = 31.59, *p* = .04 respectively). Sheltering and Similarities scores were more positive for both undergraduates and postgraduates compared to those educated to age 18 (Sheltering: χ^2^ = 54.93, *p*< .001 and χ^2^ = 50.19, *p* = .001 respectively; Similarities: χ^2^ = -53.91, *p*< .001 and χ^2^ = -33.85, *p* = .01 respectively).

Contrary to predictions (hypothesis five), whether participants’ contact with people with intellectual disabilities was voluntary or involuntary did not affect explicit attitudes: Empowerment: *U* = 3996, z = -1.74, *p* = .08; Sheltering: *U* = 5110, z = 1.20, *p* = .27; Exclusion: *U* = 4677, z = -0.01, *p* = .99; and Similarities: *U* = 4098, z = -1.56, *p* = .12.

Results for social distance scores were mixed. In line with the predictions (hypothesis five), men reported a greater desire for social distance than women on the IDLS (*U* = 14 526, z = 3.60, *p* < .001) and MASMR social distance scales (*U* = 14 103, z = 3.10, *p* = .002) Furthermore, Kruskall-Wallis analyses indicated that MASMR social distance scores differed by educational attainment (χ^2^ = 7.25, *p* = .03), with participants educated up to age 18 years desiring more social distance than postgraduates (χ^2^ = 35.31, *p* = .03). Contrary to predictions (hypothesis five), a Kruskall-Wallis test showed no significant differences in IDLS social distance scores (χ^2^ = 2.11, *p* = .35) for participants with varying educational attainments and no differences in IDLS (*U* = 5135.50, z = 1.16, *p* = .25) or MASMR social distance (*U* = 4750, z = 0.18, *p* = .86) scores between individuals with voluntary and involuntary contact, see Table 7.

## Discussion

This study investigated implicit and explicit attitudes, desire for social distance, emotional reactions towards and contact with individuals with intellectual disabilities among a UK sample. The key results can be summarised as follows: 1) we found no associations between implicit attitudes and explicit attitudes or social distance; explicit attitudes were associated with social distance. 2) Contrary to predictions, emotional reactions and implicit attitudes were not correlated, but instead there were small to moderate correlations between emotional reactions, explicit attitudes and social distance. 3) Implicit attitudes did not vary by contact, gender or educational attainment. Explicit attitudes were influenced by prior contact, but not its amount or the type of contact relationship; gender; and educational attainment. Social distance scores varied according to amount of contact, gender and educational attainment but not by type of relationship.

The lack of associations between implicit and explicit attitudes and implicit attitudes and social distance were expected. Previous research found only small to moderate associations between implicit and explicit attitudes towards other social groups (e.g. racial groups or homosexuals) [59]. Of more interest though are the differences between self-reported explicit attitudes and the more unconscious attitudes measured via the ST-IAT. Overall, this sample showed particularly positive explicit attitudes towards individuals with ID compared to a previous UK study [60]. Their MASMR social distance scores were similarly low as those reported for a representative Canadian general sample [18]. One may question how accurate these self-reports are given their marked contrast to the subtly negative implicit attitudes measured by the ST-IAT.

How is it that these attitudes were so disparate? In the first instance it is worth highlighting that the internal consistency of the ST-IAT and the other measures used in the study was good. As such a lack of association between these variables is unlikely to have been due largely to measurement error. A limitation here is that we did not have the opportunity to explore the test-retest reliability of the measures used, especially the ST-IAT. This may have added to the strength of this argument and further research may well wish to include multiple data collection points to allow for more detailed exploration of the psychometric properties of the ST-IAT when exploring implicit attitudes towards individuals with intellectual disabilities.

One may argue the differences relate merely to social desirability driving participants to be explicitly very positive, with implicit attitudes providing a more realistic reflection of the general dislike of this population. Some authors go further, arguing that a hostile, demeaning view of individuals with disabilities as inferior and undesirable permeates our society and serves as a backdrop to vicious hate crimes against individuals with disabilities [61–62]. Arguably if hostile, contemptuous views were held by a significant proportion of the present sample, one might have expected implicit attitudes to be far more negative. Alternatively the fact that nearly 35% of participants held moderate to strong negative implicit attitudes (of these 7.6% strong negative implicit attitudes) could be interpreted as support for Quarmby’s argument [61–62]. Further exploration of attitudes via the IAT may give further insight into deeply held views among different sections of society, something pertinent to identifying suitable targets for attempts to combat intellectual disability stigma and hate crime.

Alternatively one may argue that participants may well support the inclusion of individuals with intellectual disabilities and that their implicit attitudes reflect “aversive disablism” [63]; that is widespread negative stereotypes that prevail because people behave in subtly prejudiced ways which actually reinforce negative stereotypes, even though at face level they may believe that this population should be treated equally. An example of aversive disablism would be advocating the empowerment and inclusion of individuals with intellectual disabilities within society, but choosing not to use shared leisure facilities. Another possibility suggested by Deal [63], in line with work on aversive racism [64], is that people may not be anti-disabilities per se (i.e. anti-out-group), but in fact more pro-in-group. Nonetheless, pro-in-group attitudes are seen to be as damaging as anti-out-group attitudes [64].

What is the likely impact of the attitudes encountered in this study on behaviour? Meta-analytic data suggests that implicit attitudes have moderate predictive validity of an individual’s behaviour [65]. Behaviours not consciously controllable, e.g. eye contact or body language, are more influenced by implicit attitudes whereas more deliberate behaviours, e.g. verbal interactions, are more influenced by explicit attitudes [12]. Furthermore, explicit attitudes are likely to influence behaviour when individuals have either the motivation or time to consider the consequences of their actions. However, when time is constrained or motivation low, implicit attitudes are more likely to influence behaviour [12]. Therefore, although the positive explicit attitudes observed in this study may predict fairly positive deliberate behaviour towards individuals with intellectual disabilities, the negative implicit attitudes observed may drive subtly prejudiced non-verbal behaviours, interfering with the formation of positive social relations.

The lack of association between implicit attitudes and emotional reactions was unexpected. The emotional reactions measured may only apply to the specific individual described in the vignette rather than individuals with intellectual disabilities in general (which is what the ST-IAT was measuring). Alternatively, unconsciously held ideas about individuals with intellectual disabilities may have had a poor fit with the person described in the vignette. Furthermore, as suggested in the Value Account model [15], implicit attitudes are expressed via the affective system and only when affective responses are explicitly evaluated may they have an influence on explicit attitude formation. As the ERMIS preceded measures of explicit attitudes and social distance, emotional reactions generated by the vignette may have been appraised and used to inform responses to the subsequent attitude and stigma questions. The lack of association of emotional reactions with implicit attitudes but significant associations with explicit attitudes and social distance would then be plausible, as the vignette would have informed responses to the self-report measures but not to the ST-IAT.

In addition, in the absence of any suitable measure to assess emotional reactions to individuals with intellectual disabilities we used the ERMIS, designed to measure emotional reactions towards individuals with mental health problems. Thus the measure may have had a poor fit for the current purpose. Since completion of this study, the Attitudes to Intellectual Disabilities scale (ATTID) [66] has been published, which looks promising as it assess the three components of attitudes (cognition, affect, behaviour) and assesses emotional reactions in response to two vignettes (one portraying someone with a mild intellectual disability, the other someone with a severe intellectual disability).

Against expectations, implicit attitudes did not vary according to frequency of contact with individuals with intellectual disabilities. Given that most participants who reported prior contact only reported infrequent contact, very small numbers of participants fell into the other contact frequency categories. The small cell sizes may have resulted in a failure to detect the predicted relationship between these variables. Of note, the pattern of implicit attitudes was interesting, suggesting that as contact increases implicit attitudes become more positive, up to the point of daily contact where attitudes become more negative, resembling those of individuals with no or only infrequent contact. This merits further investigation. More negative implicit attitudes among those in daily contact may be evoked by involvement in some form of direct support or caring role. Research has identified that carers of individuals with intellectual disabilities often experience significant levels of stress and strain [67–69]. Thus their implicit attitudes may be more in line with negative, stereotyped attitudes towards this population. Using the IAT to explore this further could have significant implications for understanding the relationship between individuals with intellectual disabilities and their carers / supports and might signal areas for concern.

There were no significant differences in implicit attitudes according to gender, education level and type of contact relationship. This contrasts with previous research into explicit attitudes where women and more highly educated people often express more positive attitudes towards this population [27, 70–73]. Implicit attitudes may be more stable and less affected by respondent characteristics, whereas explicit evaluations may be subject to consciously accessible knowledge being appraised and thus influencing reactions, e.g. those with higher educational attainments may be more aware that advocating for inclusion is desirable and thus more likely to endorse such views.

The results obtained may appear disheartening. Over the last few decades considerable effort has been made to tackle negative stereotypes and attitudes held towards people with intellectual disabilities. This study highlights that, despite all these efforts, negative attitudes, in the form of implicit attitudes, appear to prevail. As research suggests that implicit attitudes influence non-verbal behaviours [12], it is likely that the negative implicit attitudes account for the negative experiences reported by individuals with intellectual disabilities, as do extremely hostile attitudes among a small minority. Further research into implicit attitudes towards this population is therefore imperative, particularly with the aim of identifying factors which may influence and change them.

### Limitations

The web based recruitment strategy was essential to use the IAT, which is computer administered, and in offering complete anonymity was deemed highly suitable to limit socially desirable responding of self-reported attitudes [74]. Women and graduates were over-represented and members of ethnic minority communities were underrepresented, which may limit generalisability. The method of participant recruitment (i.e. snowballing) which initiated participants originally through the first author’s professional and social contacts may have contributed to this. The contacts were largely from highly educated backgrounds and were younger in age. These individuals’ professional and social contacts onto which they would have sent further invitations to participate in the study were likely to have been similar in age and educational attainment. This may have limited the opportunity for a more varied sample overall. It is of note however that internet forum websites were used to try and invite other potential participants to the study who were not connected to the first author directly. A wide range of internet forums were approached to increase the chance of a varied participant pool. The forum websites were not specific to disability as this might have biased the results. Given this possible source of bias, it is of note though that implicit attitudes did not differ by gender or educational attainment.

A further point linked to sampling is that this study only explored the implicit attitudes of adults i.e. individuals over the age of 18 years. The implicit attitudes held by individuals younger in age may well have varied and this needs to be noted. Future research may well consider exploring and comparing the implicit attitudes of children / young people vs those of adults.

The possible effect of the words used in the ST-IAT on implicit attitudes also merits consideration. Although previous research suggests that category labels are not affected when both positively and negatively valenced words are included in a category [75], others [76] provide evidence suggesting that other stimulus characteristics may influence the IAT and its results. This research suggests that providing individuating information can override stereotyped attitudes in the IAT, e.g. using names of well-liked black individuals and much disliked white individuals reduces the typical pro-white bias [76]. Although the words in this study did not provide individuating information, some (e.g. “dependent”), were particularly value laden. That said, the average implicit attitude reported was far less negative than may have been expected and if the negative valence of the words was to have had an impact, implicit attitudes would likely have been far more negative. To further evaluate this, future research may compare different ST-IATs, e.g. one using only terms referring to individuals with intellectual disabilities (e.g. “mental handicap”, “cognitive impairment”, “special needs”) and one using only terms referring to characteristics (e.g. “impaired”, “dependent”).

It is also worth considering the critique that has been put forward of the evaluation criteria of the ST-IAT scores (see Table 2). Branton and colleagues [53] suggest that these criteria are somewhat arbitrary and that they do not provide a particularly meaningful interpretation of the results collected. The results reported here should therefore be considered with this in mind. Future researchers may helpfully explore these evaluation criteria more rigorously and specifically in relation to implicit attitudes towards individuals with intellectual disabilities. Suggestions from Branton et al. [53] include relating specific IAT scores to observable criteria such that the preferences assessed can be based on a more meaningful scale. Researchers may explore behaviours towards individuals with intellectual disabilities and relate this to the implicit preferences measured via the ST-IAT to ensure that the evaluative criteria are representative of the implicit preferences explored.

Lastly, upon reflection, it was noted that some of the words included in the “unpleasant” category may have also been associated with the category “disease”. Previous research [77] found the category “disability” was more likely to be associated with the category “disease” than “health”. Some of the words in the “unpleasant” category may have therefore been tapping associations between intellectual disability and disease. Future research should employ different words to explore degree of like and dislike towards this population, and/or explore the extent to which intellectual disability is associated with disease.

## Conclusions

This study provides insight into the implicit attitudes of lay people in the UK towards individuals with intellectual disabilities. It is concerning, yet in some ways predictable, that these were somewhat negative and not in line with the very positive explicit attitudes reported. Future research should examine implicit attitudes towards this population further, as well as factors which contribute to implicit attitudes and thus ways in which they may be improved.

## Supporting Information

S1 Appendix Vignette used for IDLS Social Distance subscale and ERMIS. (DOCX)

## References

[1] Emerson E, Malam S, Davies I, Spencer K. Adults with learning difficulties in England 2003/4 Department of Health 2005 Available: http://webarchive.nationalarchives.gov.uk/20130107105354/http://www.dh.gov.uk/prod_consum_dh/groups/dh_digitalassets/@dh/@en/documents/digitalasset/dh_4119944.pdf

[2] Staniland L. Public perceptions of disabled people Office for Disability Issues 2011 Available: https://www.gov.uk/government/uploads/system/uploads/attachment_data/file/325989/ppdp.pdf

[3] Abbott S, McConkey R. The barriers to social inclusion as perceived by people with intellectual disabilities. Journal of Intellectual Disabilities. 2006;10(3): 275–287. 16916851 10.1177/1744629506067618

[4] Cummins RA, Lau ALD. Community integration or community exposure? A review and discussion in relation to people with intellectual disabilities. Journal of Applied Research in Intellectual Disabilities. 2003;16(2): 145–157.

[5] Forrester-Jones R, Carpenter J, Coolen-Schrijner P, Cambridge P, Tate A, Beecham J, et al The social networks of people with intellectual disability living in the community 12 years after resettlement from long-stay hospitals. Journal of Applied Research in Intellectual Disabilities. 2006;19(4): 285–295.

[6] Eagly AH, Chaiken S. Attitude structure and function In: Gilbert DT, Fiske ST, Lindzey G, editors. The Handbook of Social Psychology. 4th ed. New York: McGraw-Hill; 1998 pp. 269–322.

[7] Prestwich A, Kenworthy J, Wilson M, Kwan-tat N. Differential relations between two types of contact and implicit and explicit racial attitudes. British Journal of Social Psychology. 2008;47(4): 575–588. 18158859 10.1348/014466607X267470

[8] Chaiken S. Heuristic versus systematic processing and the use of source versus message cues in persuasion. Journal of Personality and Social Psychology, 1980;39: 752–766.

[9] Brewer MB. A dual process model of impression formation In: Srull T K, Wyer R S Jr., editors. Advances in social cognition. Hillsdale, NJ: Erlbaum; 1988 pp. 1–36.

[10] Trope Y. Identification and inferential processes in dispositional attribution. Psychological Review, 1986;93: 239–257.

[11] Stack F, Deutsch R. Reflective and impulsive determinants of social behaviour. Personality and Social Psychology Review, 2004; 8(3): 220–247. 15454347 10.1207/s15327957pspr0803_1

[12] Dovidio JF, Kawakami K, Gaertner SL. Implicit and explicit prejudice and interracial interaction. Journal of Personality and Social Psychology. 2002;82(1): 62–68. 11811635 10.1037//0022-3514.82.1.62

[13] Bohner G, Dickel N. Attitudes and attitude change. Annual review of psychology, 2011;62: 391–417. 10.1146/annurev.psych.121208.131609 20809791

[14] Karpinski A, Steinman RB, Hilton JL. Attitude importance as a moderator of the relationship between implicit and explicit attitude measures. Personality and Social Psychology Bulletin, 2005;31: 949–962. 15951366 10.1177/0146167204273007

[15] Betsch T, Plessner H, Schallies E. The value-account model of attitude formation In: Maio GR, Haddock G, editors. Contemporary perspectives on the psychology of attitudes. Hove: Psychology Press; 2004 pp. 251–273.

[16] Bogardus ES. Social distance Yellow Springs, OH: The Antioch Press; 1959.

[17] Jorm AF, Oh E. Desire for social distance from people with mental disorders. Australian and New Zealand Journal of Psychiatry. 2009;43(3): 183–200. 10.1080/00048670802653349 19221907

[18] Ouellette-Kuntz H, Burge P, Brown HK, Arsenault E. Public attitudes towards individuals with intellectual disabilities as measured by the concept of social distance. Journal of Intellectual Disability Research. 2010;23(2): 132–142.

[19] Scior K, Addai-Davies J, Kenyon M, Sheridan JC. Stigma, public awareness about intellectual disability and attitudes to inclusion among different ethnic groups. Journal of Intellectual Disability Research. 2013;57(11): 1014–1026. 10.1111/j.1365-2788.2012.01597.x 22845699

[20] Allport G. The nature of prejudice Reading, MA: Addison-Wesley; 1954.

[21] Corrigan PW, Morris SB, Michaels PJ, Rafacz JD, Rüsch N. Challenging the public stigma of mental illness: A meta-analysis of outcome studies. Psychiatric Services. 2012;63(10): 963–973. 10.1176/appi.ps.201100529 23032675

[22] Islam MR, Hewstone M. Dimensions of contact as predictors of intergroup anxiety, perceived out-group variability, and out-group attitude: An integrative model. Personality and Social Psychology Bulletin. 1993;19(6): 700–710.

[23] Pettigrew TF, Tropp LR. A meta-analytic test of intergroup contact theory. Journal of Personality and Social Psychology. 2006;90(5): 751–783. 16737372 10.1037/0022-3514.90.5.751

[24] Gill F, Stenfert Kroese B, Rose J. General practitioners' attitudes to patients who have learning disabilities. Psychological Medicine. 2002;32(8): 1445–1455. 12455943 10.1017/s0033291702006608

[25] Slevin E. Student nurses' attitudes toward people with learning disabilities. British Journal of Nursing. 1995;4(13): 761–766. 7655263 10.12968/bjon.1995.4.13.761

[26] Slevin E, Sines D. Attitudes of nurses in general hospital towards people with learning disabilities: influences of contact, and graduate–non-graduate status, a comparative study. Journal of Advanced Nursing. 1996;24(6): 1116–1126. 8953346 10.1111/j.1365-2648.1996.tb01016.x

[27] Yazbeck M, McVilly K, Parmenter TR. Attitudes toward people with intellectual disabilities: An Australian perspective. Journal of Disability Policy Studies. 2004;15(2): 97–111.

[28] Swain J, Lawrence P. Learning about disability: Changing attitudes or challenging understanding? In: French S, editor. On equal terms: Working with disabled people. Oxford: Butterworth-Heinemann Ltd; 1994 pp. 87–102.

[29] Binder J, Zagefka H, Brown R, Funke F, Kessler T, Mummendey A, et al Does contact reduce prejudice or does prejudice reduce contact? A longitudinal test of the contact hypothesis among majority and minority groups in three European countries. Journal of Personality and Social Psychology. 2009;96(4): 843–856. 10.1037/a0013470 19309206

[30] Turner RN, Hewstone M, Voci A. Reducing explicit and implicit prejudice via direct and extended contact: The mediating role of self-disclosure and intergroup anxiety. Journal of Personality and Social Psychology. 2007;93(3): 369–388. 17723054 10.1037/0022-3514.93.3.369

[31] Sirlopú D, González R, Bohner G, Siebler F, Ordóῆez G, Millar A, et al Promoting positive attitudes toward people with Down syndrome: The benefit of school inclusion programs. Journal of Applied Social Psychology. 2008;38(11): 2710–2736.

[32] Jasinskaja-Lahti I, Mähӧnen TA, Leibkind K. Ingroup norms, intergroup contact and intergroup anxiety as predictors of the outgroup attitudes of majority and minority youth. International Journal of Intercultural Relations. 2010;35(3): 346–355.

[33] Turner RN, Hewstone M, Voci A, Vonofakou C. A test of the extended intergroup contact hypothesis: The mediating role of intergroup anxiety, perceived ingroup and outgroup norms, and inclusion of the outgroup in the self. Journal of Personality and Social Psychology. 2008;95(4): 846–860. 10.1037/a0011434 18808263

[34] Angermeyer MC, Holzinger A, Matchinger H. Emotional reactions to people with mental illness. Epidemiolgia e Psichiatria Sociale. 2010;19(1): 26–32. 10.1017/s1121189x00001573 20486421

[35] Bromley J, Emerson E. Beliefs and emotional reactions of care staff working with people with challenging behaviour. Journal of Intellectual Disability Research. 1995;39(4): 341–352. 7579992 10.1111/j.1365-2788.1995.tb00526.x

[36] Jones C, Hastings RP. Staff reactions to self-injurious behaviours in learning disability services: Attributions, emotional responses and helping. British Journal of Clinical Psychology. 2003;42(2): 189–203. 12828807 10.1348/014466503321903599

[37] Rose D, Horne S, Rose JL, Hastings RP. Negative emotional reactions to challenging behaviour and staff burnout: Two replication studies. Journal of Applied Research in Intellectual Disabilities. 2004;17(4): 219–223.

[38] Antonak RF, Livneh H. Measurement of attitudes towards persons with disabilities. Disability and Rehabilitation. 2000;22(5): 211–224. 10813560 10.1080/096382800296782

[39] Antonak R, Livneh H. The measurement of attitudes toward people with disabilities: Methods, psychometrics, and scales Springfield IL: Charles C. Thomas 1988.

[40] Hergenrather K, Rhodes S. Exploring undergraduate student attitudes toward persons with disabilities: Application of the disability social relationship scale. Rehabilitation Counseling Bulletin, 2007;50(2), 66–75

[41] Gaebel W, Zaske H, Baumann AE, Klosterkötter J, Maier W, Decker P, et al Evaluation of the German WPA ‘‘Program against stigma and discrimination because of schizophrenia Open the Doors”: results from representative telephone surveys before and after three years of anti-stigma interventions. Schizophrenia Research, 2008;98(1–3): 184–193. 17961985 10.1016/j.schres.2007.09.013

[42] White MJ, Jackson V, Gordon P. Implicit and explicit attitudes toward athletes with disabilities. Journal of Rehabilitation, 2006;72(3): 33–40.

[43] Thomas A, Vaughn D, Doyle A. Implementation of a computer based Implicit Association Test as a measure of attitudes toward individuals with disabilities. Journal of Rehabilitation, 2007;73(2): 3–14.

[44] Rojahn J, Komelasky KG, Man M. Implicit attitudes and explicit ratings of romantic attraction of college students towards opposite-sex peers with physical disabilities. Journal of Developmental and Physical Disabilities, 2008;20(4): 389–397.

[45] Greenwald AG, McGhee DE, Schwartz JLK. Measuring individual differences in implicit cognition: The implicit association test. Journal of Personality and Social Psychology. 1998;74(6): 1646–1480. 9654756 10.1037//0022-3514.74.6.1464

[46] Karpinski A, Steinman RB. The single category implicit association test as a measure of implicit social cognition. Journal of Personality and Social Psychology. 2006;91(1): 16–32. 16834477 10.1037/0022-3514.91.1.16

[47] Wigboldus DHJ, Holland RW, van Knippenberg A. Single target implicit associations. Unpublished manuscript. 2004.

[48] Bluemke M, Friese M. Reliability and validity of the Single-Target IAT (ST-IAT): Assessing automatic affect towards multiple attitude objects. European Journal of Social Psychology, 2008;38: 977–997.

[49] Harwood J, Hewstone M, Paolini S, Voci A. Grandparent-grandchild contact and attitudes toward older adults: Moderator and mediator effects. Personality and Social Psychology Bulletin. 2005;31(3): 393–406. 15657454 10.1177/0146167204271577

[50] Lane KA, Banaji MR, Nosek BA, Greenwald AG. Understanding and using the implicit association test: IV. What we know (so far) In: Wittenbrink B, Schwarz NS, editors. Implicit measures of attitudes: Procedures and controversies. New York: Guilford Press; 2007 pp. 59–102.

[51] Greenwald AG, Nosek BA, Banaji MR. Understanding and using the implicit association test: I. An improved scoring algorithm. Journal of Personality and Social Psychology. 2003;85(2): 197–216. 12916565 10.1037/0022-3514.85.2.197

[52] Nosek BA, Greenwald AG, Banaji MR. The implicit association test at age 7: A methodological and conceptual review In: Bargh J A, editor. Automatic processes in social thinking and behaviour. New York: Psychology Press; 2007 pp. 265–292.

[53] Blanton H, Jaccard J. Arbitrary metrics in psychology. American Psychologist, 2006;61(1): 27–41. 16435974 10.1037/0003-066X.61.1.27

[54] Henry D, Keys CB, Jopp D, Balcazar F. The Community Living Attitudes Scale: Development and psychometric properties. Mental Retardation. 1996a;34(3): 149–158. 8684283

[55] Scior K, Furnham A. Development and validation of the Intellectual Disability Literacy Scale for assessment of knowledge, beliefs and attitudes to intellectual disability. Research in Developmental Disabilities. 2011;32(5): 1530–1541. 10.1016/j.ridd.2011.01.044 21377320

[56] Harth R. Attitudes toward minority groups as a construct in assessing attitudes towards the mentally retarded. Education and Training of the Mentally Retarded. 1971;6(4): 142–147.

[57] Angermeyer MC, Matschinger H. Public beliefs about schizophrenia and depression: similarities and differences. Social Psychiatry Psychiatric Epidemiology. 2003;38(9): 526–534. 14504738 10.1007/s00127-003-0676-6

[58] Connolly T, Williams J, Scior K. The effects of symptom recognition and diagnostic labels on public beliefs, emotional reactions and stigma associated with intellectual disability. American Journal on Intellectual and Developmental Disabilities. 2013;118(3): 211–223.59. 10.1352/1944-7558-118.3.211 23734616

[59] Hofmann W, Gawronski B, Gschwender T, Le H, Schmitt M. A meta-analysis on the correlation between the Implicit Association Test and explicit self-report measures. Personality and Social Psychology Bulletin. 2005;31(10): 1369–1385. 16143669 10.1177/0146167205275613

[60] Scior K, Kan K, McLoughlin A, Sheridan J. Public attitudes toward people with intellectual disabilities: A cross-cultural study. Intellectual and Developmental Disabilities. 2010;48(4): 278–289. 10.1352/1934-9556-48.4.278 20722478

[61] Quarmby K. Getting away with murder: Disabled people’s experiences of hate crime in the UK. Scope. 2008 Available: http://www.scope.org.uk/Scope/media/Images/Publication%20Directory/Getting-away-with-murder.pdf?ext=pdf

[62] Quarmby K. Scapegoat: Why we are failing disabled people Portobello Books: London; 2011.

[63] Deal M. Aversive disablism: Subtle prejudice toward disabled people. Disability and Society. 2007;22(1): 93–107.

[64] Gaertner SL, Dovidio JF. Reducing intergroup bias: the common ingroup identity model Hove: Psychology Press; 2000.

[65] Greenwald AG, Poehlman TA, Uhlmann EL, Banaji MR. Understanding and using the Implicit Association Test III: Meta-analysis of predictive validity. Journal of Personality and Social Psychology. 2009;97(1): 17–41. 10.1037/a0015575 19586237

[66] Morin D, Crocker AG, Beaulieu-Bergeron R, Caron J. Validation of the attitudes toward intellectual disability–ATTID questionnaire. Journal of Intellectual Disability Research. 2012;57(3): 268–278. 10.1111/j.1365-2788.2012.01559.x 22533629

[67] Dillenburger K. ‘How long are we able to go on?’ Issues faced by older family caregivers of adults with disabilities. British Journal of Learning Disabilities. 2011;39(1): 29–38.

[68] Hatton C, Brown R, Caine A, Emerson E. Stressors, coping strategies and stress-related outcomes among direct care staff in staffed houses for people with learning disabilities. Journal of Applied Research in Intellectual Disabilities. 1995;8(4): 252–271.

[69] Kenny K, McGilloway S. Caring for children with learning disabilities: An exploratory study of parental strain and coping. British Journal of Learning Disabilities. 2007;35(4): 221–228.

[70] Akrami N, Ekehammar B, Claesson M, Sonnander K. Classical and modern prejudice: Attitudes toward people with intellectual disabilities. Research in Developmental Disabilities. 2006;27(6): 605–617. 16309887 10.1016/j.ridd.2005.07.003

[71] Antonak RF, Mulick JA, Kobe FH, Fiedler CR. Influence of mental retardation severity and respondent characteristics on self-reported attitudes toward mental retardation and eugenics. Journal of Intellectual Disability Research. 1995;39(4): 316–325. 7579989 10.1111/j.1365-2788.1995.tb00523.x

[72] Burge P, Ouellette-Kuntz H, Lysaght R. Public views on employment of people with intellectual disabilities. Journal of Vocational Rehabilitation. 2007;26(1): 29–37.

[73] Esterle M, Sastre MT, Mullet E. Judging the acceptability of sexual intercourse among people with learning disabilities: French laypeople's viewpoint. Sexuality and Disability. 2008;26(4): 219–227.

[74] Joinson A. Social desirability, anonymity, and internet-based questionnaires. Behavior Research Methods, Instruments, and Computers. 1999;31(3): 433–438. 10502866 10.3758/bf03200723

[75] De Houwer J. A structural and process analysis of the Implicit Association Test. Journal of Experimental and Social Psychology. 2001;37(6): 443–451.

[76] Govan CL, Williams KD. Changing the affective valence of the stimulus items influences the IAT by re-defining the category labels. Journal of Experimental Social Psychology. 2004;40(3): 357–365.

[77] Park JH, Faulkner J, Schaller M. Evolved disease-avoidance processes and contemporary anti-social behaviour: prejudicial attitudes and avoidance of people with physical disabilities. Journal of Nonverbal Behavior. 2003;27(2): 65–87.

