# Supplementary material for: Implicit Attitudes towards People with Intellectual Disabilities: Their Relationship with Explicit Attitudes, Social Distance, Emotions and Contact
Source: PLoS One. 2015 Sep 14;10(9):e0137902. doi: 10.1371/journal.pone.0137902 (PMC4569292; doi:10.1371/journal.pone.0137902)
Supplement: S1 Appendix — (DOCX) [file pone.0137902.s001.docx]

# Supporting Information

**S1 Appendix.** Vignette used for IDLS Social Distance subscale and ERMIS

*James is 32 and lives at home with his parents. James has a learning disability (mental handicap). He attended a special school. James has never had a job. He is supported by his parents with everyday activities such as caring for himself and preparing meals, as he finds it difficult to do these things on his own. He enjoys going to the park to play football, as well as visiting his siblings and going to the cinema. He attends a centre run by Mencap on a Wednesday where he enjoys activities such as art. On a Friday evening he goes to a social club for people with learning disabilities with a support worker. He is able to communicate through talking, but finds complicated or abstract language or ideas difficult. He sometimes uses pictures to help others to understand him and likes interacting with others including people he meets in the street and on buses.*
